# Results in keratoconus correction with Kerasoft 3 Contact lenses

**Published:** 2020

**Authors:** Cristina Nicula, Dorin Nicula, Corina Suciu

**Affiliations:** * Department of Ophthalmology, “Iuliu Hațieganu” University of Medicine and Pharmacy, Cluj- Napoca, Romania; ** Oculens Clinic, Cluj-Napoca, Romania; *** County Eye Hospital Cluj-Napoca, Romania

**Keywords:** contact lenses, Kerasoft 3, keratoconus

## Abstract

**Introduction:** The purpose of the paper was to evaluate the indications, fitting, advantages and functional results of Kerasoft 3 contact lenses in keratoconus.

**Material and method:** A retrospective single center study was performed at Oculens Private Clinic in Cluj-Napoca, Romania. Our study included 61 eyes of 35 patients diagnosed with keratoconus in different stages of evolution fitted with Kerasoft 3 lens. The study was undergone between August 2015 and January 2019.

**Results:** In our study, the mean age of the patients was 26.36±8.69 years. The group of study included 80.32% males and 19.62% females. Regarding previous surgeries, CXL was performed in 25 eyes, ICR in 1 eye, CXL and ICR in 15 eyes. The mean BCVA habitual was 0.38±0.19 logMar and with the lens 0.22±0.23 logMar (p<0.01). Spherical equivalent (SE) at baseline was -5.78 and after fitting the lens it decreased to -0.46. Comfort and tolerance level were maximum in all cases. No significant complications were noted with the use of contact lens.

**Conclusions:** Kerasoft 3 contact lenses provide many of the benefits of RGP lenses (avoiding RGP’s discomfort and allergic reactions), along with excellent comfort, visual acuity, high oxygen permeability and longer wearing times.

**Abbreviations:** CXL = cross-linking; ICR = intrastromal corneal ring; BCVA = best corrected visual acuity; SE = spherical equivalent; RGP = rigid gas permeable contact lenses

## Introduction

Keratoconus (Kc) is a corneal progressive and degenerative disorder that appears in the second to the third decade of life, characterized by a conical shape of the cornea (thinning, ectasia) inducing an irregular astigmatism, myopia and corneal protrusion. In late stages, corneal scars develop.

The early management of Kc consists in the prescription of glasses, contact lenses (CL) and cross-linking–UVA therapy (CXL) in order to stop or arrest the progression of the disease. The use of CL continues to play a major role in the management of Kc. Contact lenses include the rigid ones (Rigid gas permeable), soft spherical and toric silicone hydrogel, scleral and piggyback. In late stages, intracorneal rings (ICR), penetrating keratoplasty (PKP) and deep anterior lamellar keratoplasty (DALK) are indicated [**1**,**2**].

Soft lenses, designed specifically for Kc, have a useful role in correcting corneal irregularities in the early stages of the disease or when the patient does not tolerate RGP.

Kerasoft lenses (Ultravision, UK) are two types: conventional hydrogel and silicone hydrogel lenses. These lenses are marked with a laser sign at six o’clock position. Keratoconus and post graft fitting can be treated with the soft lens KeraSoft®3. These lenses have a toric design on the front surface and they contain 74%* Definitive™ Silicone Hydrogel. This enables them to provide a prolonged usage, better visual acuity and they are also easy to wear.

The characteristics of the lens can be seen in **Table 1**.

**Table 1:** The KeraSoft®3 lens characteristics

Material	Definitive™ Silicone Hydrogel 74% water content*
Modulus	0.38 MPa (Typical of mid water content materials)
Base Curves	Series A (8.00mm), B (8.20mm), C (8.40mm), D (8.60mm)
Diameter	14.00mm 14.50 mm 15.00mm
Lens design	Front Surface Asphere or Aspheric Toric prism ballasted with balanced overall thickness. Wavefront Aberration Control
Power Range	Sphere: +30.00DS to -30.00DS (in 0.25 steps) **, Cylinder: -0.50 to -11.00DC (in 0.25 steps), Axis: 0° to 180° (in 1° steps), Add up to +3.00 (in 0.25 steps)
Handling Tint	Clear
DK	60 x 10 ˗11 (cm2/sec)[ml02/(ml x mmHg)]
Modality	3-monthly lenses for daily wear
Pack size	Single lens, 2-pack, 4-pack

KeraSoft®3 has an aspheric anterior zone to maintain a toric design with a prism ballasting.

With wearing of KeraSoft®3 lenses, it has been demonstrated that the patients do not have as many complications as other common lenses due to the 74% water content. For this reason the lens can be more comfortable in various meteorological conditions. 

The purpose of the paper was to evaluate the indications, fitting, advantages and functional results of Kerasoft 3 contact lenses in keratoconus. 

## Material and method

A retrospective single center study was performed at Oculens Private Clinic in Cluj-Napoca, Romania. Our study included 61 eyes of 35 patients diagnosed with keratoconus in different stages of evolution fitted with Kerasoft 3 lens. The study period was between August 2015 and January 2019.

The inclusion criteria took into account: age- over 18 years old, any gender, diagnosed Kc (corneal topography) of stage 1 or 2 (according to Krumreich classification), Vogt striae, CXL or ICR performed previously and RGP in tolerance area.

The exclusion criteria included: the presence of systemic disease affecting ocular health, use of any systemic or topical drugs that could affect ocular physiology or lens performance, refractive astigmatism more than 5 D, atypical scar or neovascularization within the central 4mm of the cornea, aphakia and pregnancy or currently breast-feeding.

**Fig. 1:**
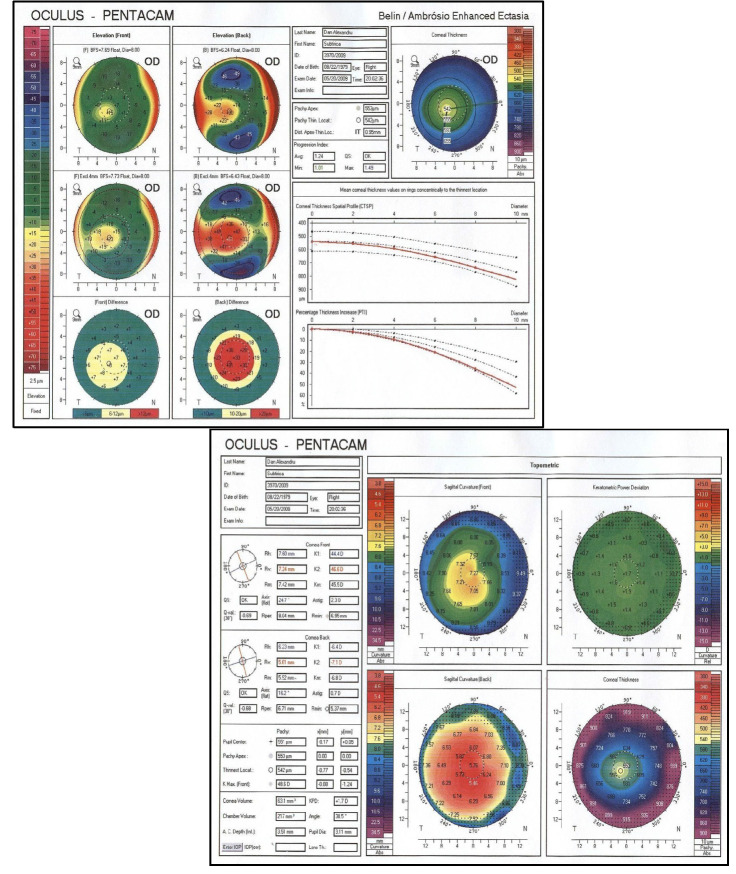
Corneal topography – keratoconus aspect

Before fitting, a complete ophthalmological examination was performed including: uncorrected and best corrected visual acuity (UCVA, BCVA), refractometry (Topconauto refracto-keratometer, KR 8900), corneal topography with pachymetry (Pentacam® HR Premium; Oculus Optikgerate GmbH, Wetzlar, Germany), keratometry and slit lamp examination (Slit Lamp BX 900, Haag-Streit AG) (**Fig. 1**).

Visual acuity was examined on Snellen charts and then for scientific purpose transformed in logMar.

Before starting fitting, we chose the proper CL for trial from the set trial consistent with keratometry and the stage of keratoconus. For early stages, we used the -2 or plan D, 8.6, 14.5 mm CL, for moderate stages we used -6/-4, 8.4, 14.5 mm CL and for advanced stages we used -10/-8D, 8.2, 14.5 mm. We left the patient 30 minutes with the contact lens on the eye and rechecked the visual acuity (VA), contact lens mobility (1-2 mm movement was acceptable), comfort and over-refraction. In cases of increased or decreased motility, we changed the CL with a smaller or a higher curvature respectively. The fitting assessment of the lens included the evaluation of the VA with the lens. Poor VA indicated a poor lens fit. After 3 months of wearing the prescribed contact lenses, we performed the reevaluation of the patient (VA, over-refraction, mobility, comfort) and prescribed the final contact lens. The follow–up period was at 6 months.

Regarding statistics, the follow up measurements made at 6 months after fitting were compared with baseline values, and statistical analysis was performed using a 2-tailed paired sample Student t test. A p-value <0.05 was considered statistically significant.

## Results

In our study, the mean age of the patients was 26.36±8.69 years. The group of study included 80.32% males and 19.62% females. CXL was performed as previous surgery in 25 eyes, ICR in 1 eye, CXL and ICR in 15 eyes.

Baseline refractive characteristics regarding the spherical equivalent, cylinder and keratometry are shown in **Table 2**.

**Table 2:** Baseline refractive characteristics

Sphere	K avg		
	<45.00	45.00-50.00	>50.00
No. of patients	14	29	21
Mean	-1.02	-3.19	-7.31
SD	2.44	2.86	4.88
Cylinder	K avg		
	<45.00	45.00-50.00	>50.00
No. of patients	14	29	21
Mean	-3.25	-3.32	-3.10
SD	1.61	1.26	0.95
Curve	K avg		
	<45.00	45.00-50.00	>50.00
No. of patients	14	29	21
Mean	8.56	8.46	8.34
SD	0.10	0.17	0.19

**Fig. 2:**
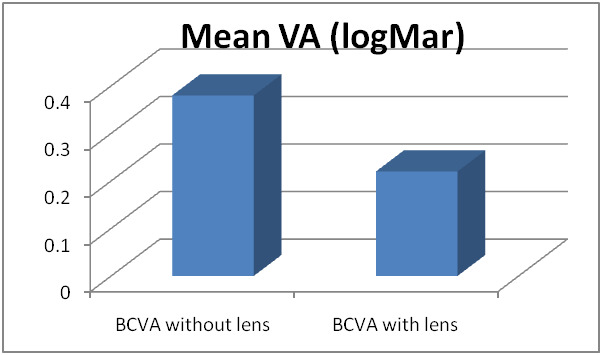
Values of BCVA habitual and with lens in logMar

Mean BCVA habitual was 0.38±0.19 logMar and with the lens 0.22±0.23 logMar (p<0.01) (**Table 3**, **Fig. 2**).

**Table 3:** Values of BCVA habitual and with lens in logMar

	BCVA habitual	BCVA with lens
Mean	0.38	0.22
STDV	0.19	0.23

**Fig. 3:**
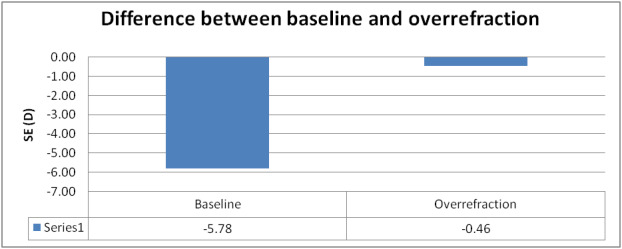
The difference of SE between baseline and over-refraction

The difference of spherical equivalent (SE) between baseline and after fitting the lens is shown in **Fig. 3**. 

The safety of these CL was established at 12 months by slit lamp examination. In most of the cases, no findings, corneal infiltrates, corneal vascularization, epithelial microcysts, bulbar congestion were observed and limbal injection grade was 0-21%. The abandon of the CL because of financial reasons was registered in three cases.

## Discussions

Contact lenses continue to play a role in the non-surgical management of Kc [**3**]. Soft lenses have a limited role in correcting corneal irregularities, offering a poor visual acuity. However, soft contact lenses designed specifically for keratoconus, such as Kerasoft 3, may be useful in the correction of mild or moderate keratoconus [**3**].

The indications of soft contact lenses are represented by: Keratoconus stage I, II, III, hard CL intolerance or after Cross linking therapy and Ferrara rings implantation.

In our study, the mean age presentation was 26.36±8.69 years. Similar data were offered by Crews et al. [**4**] in a retrospective study and found that the mean age at referral was 28 years. Seema Das et al. [**3**] noted in their study that the mean age at presentation was 25.3 years. Nevertheless, it was very difficult to establish the age onset of the disease because some of the patients paid attention to their decreased visual acuity only when both eyes were affected or when they were examined by the optometrists. There are studies that highlighted the same idea [**5**].

In our study, males were affected predominantly (80.32%). Similar outcomes were shown by several studies [**6**-**8**] during time.

Contact lenses normally offer the patient a better visual acuity in comparison with glasses, by acting against the irregular astigmatism induced by Kc. Moreover, the progression of the disease may often require the change of glasses prescription and sometimes, even with best correction, it is not possible to obtain a good visual acuity. In our study, we obtained a significantly statistical difference regarding the habitual VA and VA with the lens (p<0.01). Similar results were demonstrated by Seema Das et al. [**3**] and Frederick et al. [**9**].

Even more, in our study we took into account not only the stage of Kc but also the keratometric readings. That is why we used Kerasoft 3 in mild and moderate stages of Kc, under 50D values of the corneal curve. Seema Das et al. [**3**] used soft lenses in Kc correction in 6% of the cases. In eyes with a keratometry above 50D, studies showed that RGPs are indicated and, in advanced cases, Rose K or Kerasoft IC lenses are the proper choice [**10**,**11**].

When prescribing soft contact lens for Kc, we have to take into account not only the keratometry but also the advantages offered by the lens: simple fitting, excellent comfort, very good tolerance and good mobility.

## Conclusions

1. Kerasoft 3 CL provides many of the benefits of RGP lenses, (avoiding RGPs discomfort and allergic reactions), along with excellent comfort, visual acuity, high oxygen permeability and longer wearing times. 

2. This CL offers a solution regarding the mechanical stress of the cornea, a major factor that contributes to keratoconus.

3. The Kerasoft 3 provides a very important opportunity for ophthalmologists to overcome the many difficulties of fitting keratoconus.

**Acknowledgements**

All authors have equal contribution to this paper.

**Source of funding**

No source of funding to declare.

**Disclosures**

None.
